# Differences in antiretroviral scale up in three South African provinces: the role of implementation management

**DOI:** 10.1186/1472-6963-10-S1-S4

**Published:** 2010-07-02

**Authors:** Helen Schneider, David Coetzee, Dingie Van Rensburg, Lucy Gilson

**Affiliations:** 1 School of Public Health & Family Medicine, University of Cape Town, Anzio Road, Observatory, Cape Town, 7925, South Africa; 2 Centre for Health Systems Research & Development, University of Free State, South Africa; 3 Health Policy Unit, London School of Hygiene and Tropical Medicine, London, UK

## Abstract

**Background:**

South Africa’s antiretroviral programme is governed by defined national plans, establishing treatment targets and providing funding through ring-fenced conditional grants. However, in terms of the country’s quasi-federal constitution, provincial governments bear the main responsibility for provision of health care, and have a certain amount of autonomy and therefore choice in the way their HIV/AIDS programmes are implemented.

**Methods:**

The paper is a comparative case study of the early management of ART scale up in three South African provincial governments – Western Cape, Gauteng and Free State – focusing on both operational and strategic dimensions. Drawing on surveys of models of ART care and analyses of the policy process conducted in the three provinces between 2005 and 2007, as well as a considerable body of grey and indexed literature on ART scale up in South Africa, it draws links between implementation processes and variations in provincial ART coverage (low, medium and high) achieved in the three provinces.

**Results:**

While they adopted similar chronic disease care approaches, the provinces differed with respect to political and managerial leadership of the programme, programme design, the balance between central standardisation and local flexibility, the effectiveness of monitoring and evaluation systems, and the nature and extent of external support and programme partnerships.

**Conclusions:**

This case study points to the importance of sub-national programme processes and the influence of factors other than financing or human resource capacity, in understanding intervention scale up.

## Background

Among a number of systems constraints described by Mangham and Hanson [1] in their review of the scale up literature, health sector “policies and management” were described as both “less amenable to buy-out through the provision of additional funds”, and as inadequately researched. In a commentary on this review, two of us emphasized the central role of strategic management in scale up, at the heart of which is the capacity to engage flexibly with multiple actors and interests whilst taking account of the constraints and possibilities of the implementation environment [2]. This paper seeks to deepen understanding of these processes, with a specific focus on approaches to programme management in the scale up of antiretroviral therapy (ART) in South Africa.

In April 2004, South Africa launched an ART programme through the public health system. Prior to this, ART was provided through the private sector and local initiatives in a few dozen sites in the public health system, the most celebrated of which was the *Médicins-Sans-Frontières* (MSF) supported Khayelitsha programme in the Western Cape Province. Scale up was initially guided by a national framework, the Comprehensive Care Management and Treatment (CCMT) Plan (2003), which proposed a progressive expansion in access over five years. This was succeeded by the 2007 National Strategic Plan (NSP) which established universal access targets (defined as the annual enrolment of 80% of those newly eligible onto ART) over five years. On the basis of the CCMT Plan, initial funding for implementation of the policy was mobilised from the national budget through conditional grants, standardized first and second line drug regimens were defined, and treatment guidelines developed. Drugs were sourced through national tenders, and a centralized accreditation process for the establishment of “CCMT” sites was instituted.

According to official reports, by early 2009, roughly 700,000 people had been enrolled onto ART through the public health system, which accounts for nearly 80% of all ART provision in the country (National Comprehensive HIV and AIDS Plan Statistics, January 2009). However, in the face of overwhelming need and lagging resources, ART coverage rates are still low – in 2008 coverage was still only 38.4% of need [3].

South Africa has a quasi-federal political system with three spheres of government – national, provincial and local. Each sphere has elected political representatives and its own areas of authority. The national sphere collects revenue and sets overall policy, but devolves responsibility for implementation of most government functions to nine provincial governments. National revenue is allocated to provincial governments in one of two ways – as block grants that are allocated by provincial governments across sectors of health, education etc. (referred to as the provincial equitable share) or as conditional grants for specific purposes. Provincial HIV programmes, including ART, are funded predominantly through national conditional grants with provinces supplementing these grants from their own or donor funds to varying degrees.

Despite the presence of ring-fenced funding and specific national policy, one of the striking features of ART scale up in South Africa has been the level of provincial variation in outcomes: in 2008, ART coverage rates (using national criteria of need) ranged from 26% (Free State) to 72% (Western Cape) [3]. What accounts for this variation between provinces, given common plans and clear funding mechanisms? Possible explanations offered so far include differences in health system capacity, the size of the HIV burden and provincial political contexts [4][5]. While these factors, alone or in combination, are relevant to understanding variation, they do not sufficiently explain this variation. This paper focuses on the role of provincial programme implementation as a set of political and managerial choices further influencing programme scale up.

Implementation and implementation research is emerging as a special area of interest in the public health field. The gap between policy objectives and policy outcomes has prompted increasing global concern with improving health policy implementation [6], and a growing realization that the availability of cost-effective interventions does not automatically ensure their uptake in health systems and professional practice [7].

In this context, implementation is the process by which policy decisions, plans and programmes are operationalised through health systems. Brinkerhoff and Crosby [8] have spelt out the nature of managerial actions required for successful policy implementation, which, they argue, lie along a continuum from operating to strategic tasks. The particular mix of operating/strategic actions depends on the scale and complexity of change, which varies depending on whether the implementation involves a project, a programme, or a policy. At the project implementation end of the spectrum operational tasks include setting clear objectives (targets), defining roles and responsibilities and establishing feedback (monitoring) mechanisms. Programme implementation introduces more strategic dimensions such as the need for active leadership, programme design and ensuring collaboration between multiple groups and organizations. The most complex management tasks, with the greatest strategic content, are associated with policy implementation: building legitimacy and constituencies for change, mobilizing resources, and organisational modification. Although the tasks of ART scale up could be regarded as essentially programmatic in nature, both the scale of the HIV epidemic and the ART programme make these strategic managerial tasks particularly relevant.

Amongst these facets of implementation, recent writers on the health system as a complex organisation have drawn attention to the dimension of programme design and its influence on implementation. They challenge the conventional view that the more detailed the design of programmes, the more standardized their implementation. Instead, they argue, “creative progress towards a difficult goal can emerge from a few flexible, simple rules, or so called minimum specifications" [9: 324]. Minimum specifications (or core rules) provide overall direction and the boundaries of action but also promote innovation and collaborative networks at all levels. The most effective course of action emerges with time through experimentation and learning during which innovations are adapted locally in an active process of achieving an “intervention-system fit” [10]. It follows that opportunities for initial “trialling” of interventions on a smaller scale, the co-option of reflective front-line practitioners with tacit, insider knowledge on how to manoeuvre in the system and the presence of strong collaborative networks – both formal and informal – greatly enhance implementation.

Drawing broadly on Brinkerhoff and Crosby's typology, as well as the recent literature on complexity, we compare and contrast operational and strategic management features of ART programme implementation in three provinces of South Africa - Western Cape, Free State and Gauteng. We discuss how these may have facilitated or constrained scale up of the programme. The three provinces represent the range of ART programme coverage, and therefore scale up success, in South Africa, from lowest (Free State) to highest (Western Cape) and average (Gauteng) coverage rates.

## Methods

The analysis makes use of operational research conducted by the authors over the past six years, as well as the observations made possible by this involvement over time. The first source of data is three “models of ART care” studies which documented patient profiles, access barriers, resources, chronic disease care systems including adherence management and degree of integration with other services of the ART programme in the three provinces. Between 2005 and 2007, using a set of similar tools, 16 ART delivery sites (Western Cape 8, Gauteng 4, Free State 4) were reviewed in depth. The sites were purposefully selected to reflect the different realities and models of ART provision. They included PHC and hospital-based, single purpose and integrated, and first-line and referral ART sites. The data collected followed a typical multi-method health service evaluation and are reported in detail elsewhere [11]: they included key informant interviews with 75 facility personnel, completion of checklists, extraction of routine data, record reviews, focus group discussions, self-administered questionnaires and just over 2,100 patient exit interviews.

The second source of data is an in-depth and longitudinal programme of work on ART implementation in the Free State Province, conducted between 2004 and 2008. Apart from documenting programme implementation in a cohort of health facilities and patients in the ART programme, the research formally tracked developments at provincial level through participant observation by one co-author [12].

Such formal assessments were not done in Gauteng and Western Cape, although two authors (HS & DC) held provincial health department appointments as public health specialists in Gauteng and Western Cape Provinces, respectively, and in this capacity conducted evaluations for the HIV programmes of the two provincial governments. This gave them a vantage point from which to observe provincial processes. These observations were complemented by published accounts of programme implementation and the political context of decision-making in these provinces, written by an array of actors that included senior programme managers [13], activists seeking change from outside the system [14] and academic observers [15][5].

In a largely inductive process, these sources were reviewed for information on provincial programme implementation. Relevant information was extracted and coded into themes and categorised into operational and strategic management actions. The paper was also read and reviewed by two HIV experts with detailed knowledge of implementation of the HIV programme in South Africa. Finally, one author (LG) was an outsider to the particular processes but knowledgeable of South Africa’s health policy process in general.

We do not claim to provide an exhaustive inventory of ART programme management tasks nor do we seek to establish causal connections in any narrow sense. Instead, following Pawson and Tilley [16], we suggest that a consideration of programme implementation can help to make sense of varying coverage. As they point out, “Evaluating in open systems is a profoundly uncertain business…[However], it should be possible to detect some processes activated within the programme that may be responsible for and make sense of the changes observed. [And] it should be possible to detect something about the conditions and circumstances in which the intervention is mounted which allow for and make sense of the observed process and outcomes.” [16:16]

It is also important to note that the analysis focuses on the early period (2004-7) of scale up in South Africa, where funding constraints were not considered a key determinant of scale up [17]. This has changed drastically since late 2008, when interruptions of supplies of ART were experienced for the first time in the Free State Province and inadequate programme financing re-emerged as a central obstacle to future scale up.

## Results

### Programme coverage and context

**Figure 1:**
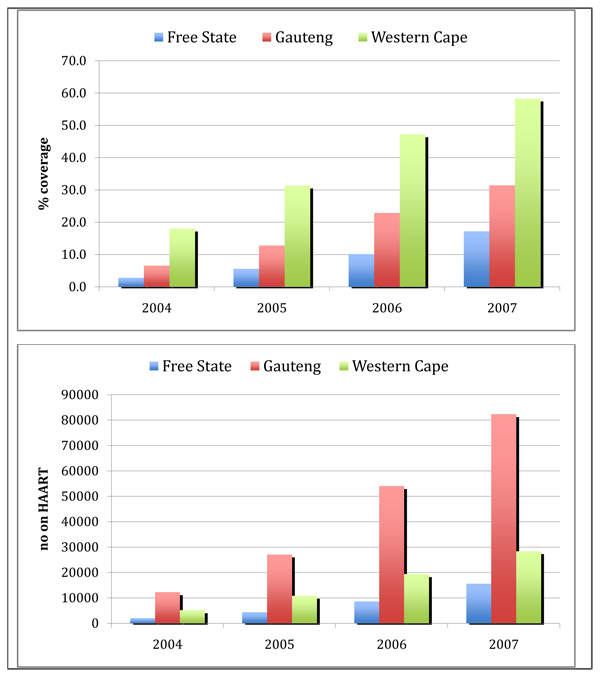
**Coverage and numbers of adults on Highly Active Antiretroviral Therapy (HAART), Free State, Western Cape and Gauteng, 2004-2007.** Source: Personal communication, Leigh Johnson, 2009

Figure 1 shows the evolution in coverage rates for ART in the three provinces, indicating a pattern of inequalities in scale up dating back to 2004. Coverage is defined as the proportion of the population in need receiving ART, where need is those with CD4<200 or with AIDS defining illness. These criteria have since been revised.

When looked at in absolute terms, programme scale up has been far more dramatic in Gauteng than in the other two provinces. In this respect, Gauteng represents the most significant scale up effort of the three provinces, involving the greatest mobilisation of resources and programmatic attention. Differences between coverage levels (in %) and absolute numbers within provinces are reflective of the lower burden of disease and smaller population in the Western Cape relative to Gauteng and Free State (Table 1)

**Table 1:** Comparison of provincial populations, HIV prevalence, human resources, and funding for HIV Programme

	Free State	Gauteng	Western Cape
Public sector dependent population (PSDP) mid 2007*	2.5 million	7.5 million	3.9 million
Population in urban settlements	75.8%	97.2%	90.4%
Antenatal HIV prevalence 2007	31.5%	30.6%	15.3%
Professional nurses per 100,000 public sector dependent population in 2007	131.6	107.3	114.0
Doctors/100,000 public sector dependent population in 2007	23.2	32.0	33.8
Spending on primary health care in 2007 (rand per capita public sector dependent population)	233	312	428
2005/6 HIV conditional grants (rand per capita public sector dependent population)	40	25	21
Additional resources mobilised for HIV programme in 2005/6 (rand per capita PSDP)	0	33(Provincial Equitable Share)	20(Global Fund + Provincial Equitable Share)

Sources: [27][28][29]

* Approximately 85% of South Africa’s population is dependent on the public health system for care

The three provinces also vary in other dimensions – Gauteng and Western Cape are highly urbanised provinces and have a greater availability of medical practitioners and higher expenditure on primary health care in the public health sector than the Free State Province. The latter, however, has a higher ratio of professional nurses per population than the other two provinces. Within the South African public health system, the Free State was considered one of the front-runners in the establishment of a district health system after the political changes of 1994 [18]. It received a considerably larger per capita allocation of the national HIV conditional grant in 2005/6 than the other two provinces, but in contrast did not mobilise any additional resources (either externally or from the provincial government budget) for the programme (Table 1). All three provinces benefited from donor support flowing to non-governmental partners, such as the Presidential Emergency Fund for AIDS Relief (PEPFAR), although in different ways (see later). Thus while broadly falling within the same national health system and policy framework, each province has its own history and profile of strengths, weaknesses and resources.

### Provincial management of implementation

Using an adapted version of operational and strategic management tasks proposed by Brinkerhoff and Crosby [8] as the organising principle, additional file 1 summarises the elements of ART programme implementation in the three provinces that were highlighted in the documents reviewed. The following operational management tasks are discussed: creating access through availability of treatment sites, staffing and training; ensuring smooth supply of drugs; development of chronic care systems within health facilities; and establishing effective information systems. The latter could be considered as key to both operational (feedback and adaptation) and strategic (monitoring) management tasks. The other strategic tasks included leadership (political and managerial/administrative); resource mobilisation; appropriate programme design; and ensuring buy-in and participation by key actors in implementation.

### Operational management in the three provinces

**Figure 2:**
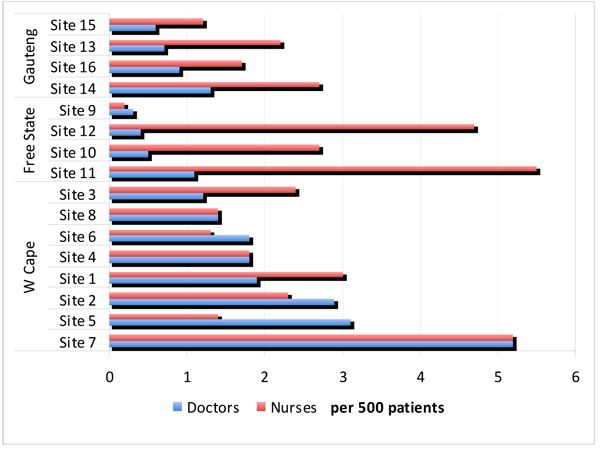
**Availability of medical and nursing personnel in 16 ART sites in three provinces, South Africa, 2005-2007** (Source: Schneider et al, 2008b)

There were a number of similarities and differences in the operational management of the ART programme in the three provinces. In terms of creating access, by the end of 2006, the Western Cape had a higher ratio of ART sites per population (Additional file 1) and availability of medical personnel (Figure 2) than the other two provinces, although Gauteng was not better served than Free State, which also had higher ratios of public sector nursing personnel (perhaps reflecting greater background availability). Both Gauteng and Western Cape had procured antiretroviral drugs prior to the finalisation of the national drug tenders (in 2004) using their own resources, in contrast to the Free State programme, which relied on a delayed and initially erratic national process. In late 2004, having finally begun its programme, the Free State experienced an interruption of antiretroviral supplies for two months following the withdrawal of certain drugs from the international market. This derailed implementation, creating an early challenge to the programme’s legitimacy in the eyes of managers, providers and patients, as it left “managers disempowered, health care workers demotivated and prospective patients disappointed as waiting lists for drugs built up.” [12:73]. The pattern of implementation in the first year of the Free State’s programme was described by one of the actors as “hurry up; slow, slow, slow; no, go, go, go.” [12:74]. Subsequent evaluations of drug availability, however, suggested uninterrupted access [19], until a budgetary crisis interrupted procurement again in 2008.

The ART sites reviewed in the models of care studies conducted in all three provinces shared a number of key characteristics:

• The adoption of contemporary chronic disease care approaches to the management of patients, that involved careful screening and treatment preparation, and social support to promote retention and adherence such as referral for disability grants, counselling, support groups and nomination of treatment supporters;

• The involvement of teams of professionals in planned pathways of care;

• Extensive recruitment and training of professional and non-professional staff.

These operational features, to some extent mandated by national guidelines, may have accounted for the high rates of self-reported adherence and viral load suppression in the early phases of scale up in all three provinces, as well as patient evaluations of ART services as more accepting and of higher quality than other services (Ibid) (Table 2).

**Table 2:** Self reported adherence and viral load suppression in patients attending 16 ART sites in three provinces (Source: Ibid)

Province	Facility No	Missed ART dose in last 3 days*		Viral load at 6 months <=400 copies/ml**	
		
		N	%	n	%
Western Cape	1	133	14	7	100
	2	183	3	141	96
	3	110	3	52	96
	4	207	7	169	94
	5	116	5	75	92
	6	110	3	138	84
	8	194	6	130	89

Free State	9	180	0	38	87
	10	79	0	32	88
	11	62	0	38	95
	12	90	0	30	97

Gauteng	13	191	1	145	89
	14	164	2	133	84
	15	194	3	117	90
	16	164	1	145	92

*Obtained in exit interviews

**Western Cape data obtained from routine facility reports; Free State and Gauteng from record reviews

Two-year retention rates in the first 12,500 people enrolled onto treatment between 2001 and 2005 in the Western Cape were 82% [20]; and in the Free State, 79% of the first 3,600 patients initiated onto HAART over a 20 month period were retained in care [21]. Although data on retention in care are not available for Gauteng Province as a whole, individual facilities surveyed for the models of care study had retention rates that varied from 69-72% [11]. Together these findings show the implementation of good quality ART programmes (if varying in coverage) in the three provinces and the ability to introduce chronic care innovations in the routine public sector environment. This was made possible by a degree of HIV exceptionalism evident in the strategic attitude to the programme in all three provinces, and its vertically-driven nature. Even though ART sites were based in existing public sector facilities and collaborated with other services, they were shielded from the rest of the system by ring-fenced resources and staffing, and managed by special implementation processes and committees.

Finally, one of the key differences between the provinces was their approach to programme monitoring. Although the national Department of Health and Treasury developed a long list of reporting indicators, they provided no specific guidance on the design of information systems for the ART programme. Poor information, especially regarding programme outcomes, has been regarded as a key weakness of South Africa’s ART programme nationally. However, a distinctive feature of the Western Cape was its high quality ART information system. It was developed through a process of consensus involving experienced TB managers (knowledgeable in the use of the TB register) and some local trialling in one of the sites (Khayelitsha) of a separate, lean, paper-based (at the point of use) system that included structured clinical records, patient cards, patient registers and cohort reporting. The ART M&E system was managed in partnership with a university which invested considerable resources in ensuring accurate data and local feedback on programme coverage, and on progress towards targets and outcomes. This provided the basis for programme review and accountability.

### Strategic management in the three provinces

The most striking differences, however, between the provinces lie in the realm of strategic management processes.

As has been well described by commentators in the field [4][5][14], the Western Cape and Gauteng Provinces benefited from open and strong political backing for the ART programme, that had already manifested around an earlier generation of policies on the prevention of mother-to-child transmission (PMTCT). In the face of ongoing ambivalence and scepticism towards the ART programme from the national political leadership, these two provinces were able to assert within the provincial bureaucracy an unambiguous imperative to expand access to ART, including providing additional resources for the programme from the provincial budget and in the case of the Western Cape, a provincial application to the Global Fund for AIDS, Tuberculosis and Malaria.

In the *Western Cape*, the political stance was driven by attitudes towards the national ruling party (African National Congress) on the part of the opposition party (Democratic Alliance) governing the province. Already in 1999, this province implemented the WHO recommendations emerging from the Thai-CDC short course AZT trial for PMTCT, and added nevirapine to the regimen when the Ugandan HIV Net012 study results were published [14]. These policies went against national recommendations and reflected the expression of provincial autonomy and legitimacy in the face of an unpopular national government position on AIDS. They were promoted by key ANC aligned officials within the bureaucracy who were willing to push implementation despite contrary pressures from the national department of health.

In *Gauteng*, political support came from an ANC-aligned provincial premier who had sufficient political capital within the ruling party to chart an independent course on AIDS in the province. The Premier was instrumental in supporting the early use of ART in this province, chaired high-level multi-sectoral provincial HIV teams and a Provincial AIDS Council, and secured a large budget for the provincial HIV programme from the provincial equitable share.

In the two provinces, provincial political commitment shielded programme implementers from national dynamics and provided room to manoeuvre in the bureaucratic spheres. It no doubt bolstered the ability of provincial ART programmes to leverage cooperation and coordination of key health sector players (districts, facilities, pharmaceutical divisions etc.) around the programme.

The *Free State* programme did not benefit from such high level backing and its officials operated within a more constrained environment, dictated by national policy and funding [12]. While seeking to express their own unique approach to the programme, their power to mobilise and influence other actors within the province was patchy. Efforts to establish an inclusive provincial Task Team to plan and manage the ART programme, involving a range of internal departmental structures and external organisations were not sustained. As Van Rensburg [12:69] describes, it became “an inwardly focused process seriously neglecting any consultation or involvement of essential partners within and beyond the FSDOH”. Most crucial was the break down in coordinated action between the ART programme and service delivery lines in the District Health System and “Clinical Health Cluster” within the Free State Department of Health. The provincial programme became increasingly vertical and isolated, plagued by a high turnover of middle level managers, preoccupied with operational rather than strategic matters, unable to deal with emerging difficulties and mired in inertia, “chronic indecision, and inaction” [12:75].

Another important difference between provinces was the choice made with respect to overall programme design. Of the three provinces, the *Free State* had the most standardised approach, the so-called “3x1 patient-walk-through” model, where patients moved between one of three “assessment” sites and a “treatment” site at particular points in the care pathway. Doctor-based services, including initiation of treatment and early follow-up occurred at a central treatment site, and pre-packaged medication was subsequently delivered to assessment sites for routine follow-up closer to people’s homes. In one more remote district, “combined sites” brought together the preparation and prescribing functions in one facility. Processes for treatment preparation, selection of treatment buddies, follow-up routines, roles of different health workers and recording of information were all clearly laid out and codified in detailed algorithms, forms, manuals and visual aids. These were designed with the assistance of external experts with the view to ensuring a carefully planned and monitored roll-out process that maximised *“quality not quantity” *[12]. However, it may have inadvertently promoted a programme style of rigidity and excessive caution, whether related to accreditation of new sites or the decision to initiate treatment in individual patients.

Moreover, a fixed approach to the model did not encourage new ideas and learning from experience or promote ownership of the programme on the part of front-line providers or district health services. One of the early problems the Free State programme was unable to address was the emerging evidence that the patient-walk-through-model, despite its intentions of promoting access and quality, was inefficient. Dividing up the care pathway between facilities disrupted continuity, produced major bottlenecks and created numerous barriers to access. The ability of the programme to resolve problems was also hampered by the absence of timely and reliable routine information. By 2006, the average time between assessment of eligibility and initiation onto ART had extended to three months in some sites [22], and the programme became characterised by high levels of mortality in those already enrolled in the programme and waiting to start ART [21].

In contrast, the *Western Cape* province’s unique programme design was based around an idea of process. As described by the architect of the programme [13], the central defining feature was that of a partnership between government and a handful of key semi-external players, who were involved in public sector service delivery from a base in a non-governmental or academic/research institution. This approach built on the experience of the PMTCT programme: “The die had been cast during the course of the PMTCT programme, mainly with the approach taken to involve partners in the implementation of the programme.” [13:250] The partners were chosen on the basis of their capacity to deploy resources and provide direct inputs into building capacity for service delivery in public health sector facilities. “Partnerships must be carefully chosen, and should ideally be well resourced, well defined, well administered and bring with them additional clinical staff.” [13:259] They included, amongst others, MSF (supporting service delivery in Khayelitsha), Absolute Return for Kids (which deployed clinical teams to kick start the ART programme in facilities until government was able to take over), referral services through an academic medicine department, and support for the M&E system from a School of Public Health.

Programme governance in the Western Cape was centered on a combined “clinician-management-partner forum” [13], which relied on the considerable chemistry between a few “leading men” in the policy community [15]. Through these processes, programme rules evolved, such as the standardisation of the information system for the province. However, individual sites were also encouraged to experiment with different approaches: “Local managers and clinical coordinators have proven to be well-informed regarding the most appropriate choices to be made and it has been possible to capitalise on this local knowledge” [13:255]. In contrast to the Free State, the Western Cape sites evaluated in the models of care study not only expressed a variety of individual delivery methods and routines, they were also more likely to modify or try out new approaches as problems were confronted and services evolved [23].

It is interesting to note that the Western Cape model was led by government but relied on direct links between senior managers and front-line providers, to some extent bypassing structures such as the district health system in the initial phases. The “partnership approach” was seen as a time bound one, with the need for “a point of closure” once the programme became established [13:259].

The *Gauteng* ART programme differed from the other two provinces in having no particular provincial design (with respect to either content or process), beyond the templates provided by national government. Apart from press releases, speeches and budget statements, there is little documentation providing a description of this programme and suggesting a distinct provincial identity or stamp. The approach appeared to be driven essentially by a provincial political mandate to expand access, with politicians and senior managers regularly making public commitments to providing resources and setting new targets towards universal access. Treatment sites were provided with the necessary inputs according to national accreditation criteria and left to decide on specific processes of care through district and facility structures. Gauteng’s approach therefore could be described as somewhat *laissez faire* - it did not specifically encourage but neither precluded pragmatic partnerships with other actors. There was thus a fair degree of variation between sites evaluated in the models of care study with respect to adherence, information and other systems, and some autonomy for local/district players to develop their own approaches to service delivery [24].

Although provincial and regional task teams brought ART site managers together on a regular basis, the Gauteng provincial government did not see the necessity of establishing joint processes of ART programme governance with non-governmental players. However, the programme implicitly drew on the experience, support and training of several large, well established and donor-funded (particularly PEPFAR-partner) ART sites based in academic centres. Much of the initial learning and growth in access to ART occurred in these sites, acting as the catalyst for the programme in the province as a whole. These sites also developed outreach and training programmes, led networks and professional forums such as the HIV Clinicians’ Society, and provided skilled and motivated staff for the newer sites. Their imprint on service provision and innovation in Gauteng was thus considerable, although their role was less formalised than in the Western Cape.

Finally, both the *Western Cape* and *Gauteng* provincial ART programmes were able to build their programmes on the “trialing” of HIV treatment sites that pre-dated the official roll-out. A national census in early 2004, just prior to the start of the national programme, counted a total of 39 ART projects across the country, predominantly in the Western Cape, Gauteng and KwaZulu-Natal [25]. When the national programme was instituted, these sites were not only able to provide a practical demonstration of “intervention-system fit”; they also established a programme style of bottom-up initiative and problem solving. In the *Free State*, on the other hand, despite the involvement of nursing and medical academics and external researchers, the programme did not have equivalent donor-funded clinical/service delivery resources of the other two provinces. It was thus designed with little grounded clinical experience to build on in the province, few local champions, weak informal networks and limited connections to other non-governmental, professional or civil society players nationally. Van Rensburg [12:66] described the programme as “a centralised, top down and unilaterally government run initiative.”

## Discussion

Despite clear national policy and funding, and a common health system framework, the implementation of the ART programme unfolded differently in the three provinces, along the following dimensions:

○ The strength of programme leadership, political and managerial/administrative, able to champion and steer the programme over time;

○ Additional resources mobilised to support implementation;

○ The ability, from within the programme, to manage and lead forms of coordinated governance with key actors involved in implementation;

○ The involvement of semi-autonomous practitioners, with sufficient power and space to innovate and problem-solve;

○ The presence of informal networks among practitioners and between practitioners and managers;

○ An appropriate balance between core rules and local latitude in implementing models of service delivery suited to local contexts;

○ The extent, nature (clinical/research/training) and funding of partnerships;

○ The ability to build on and benefit from prior learning on programme implementation;

○ The ability to establish credible mechanisms of programme accountability, including functioning information systems.

Of the three provinces, the Western Cape made the most explicit strategic managerial choices, particularly with respect to building alliances, approaches to programme design, and M&E systems. These choices can plausibly be argued to underlie differences in programme performance. However, they are unlikely to do so in isolation. An appropriate balance between local experimentation and provincial policy was enabled by a favourable political context, networks with an activist community, and the early publication of provincial successes [26]. These raised the visibility and status of the Western Cape programme which, in turn, facilitated the mobilisation of additional funds through the Global Fund. The inter-related nature of provincial histories, contexts and managerial choices in fact gave each provincial programme a distinct culture or style – in the Western Cape one of external partnerships to achieve both coverage and quality, in Gauteng a less high profile but well resourced drive to expand access, and the Free State one of uncertainty and caution.

To what extent did the Free State’s programme start from a position of disadvantage that determined its future development? The political context apparently provided it with less room to manoeuvre than Western Cape and Gauteng, although on the other hand, political leaders were not antagonistic to roll-out (as has been suggested in other provinces). It had fewer expert clinicians and less available energy from the bottom of the system, but considerable researcher involvement, and a stronger district health system foundation than other provinces. This profile provided it with specific opportunities but also possibly required greater managerial capacity from within the programme than that of the other two provinces, where politicians ensured it would be adequately resourced from the budget and prioritised by all actors. Ironically, weaker implementation contexts may require greater strategic management. Would an alternative approach that involved less focus on developing detailed programme specifications at provincial level than on fostering partnerships and greater encouragement of district ownership and decision making, have resulted in better coverage? If so, it would have necessitated a shift from a focus on operational tasks to the more actor-oriented strategic functions. Such a shift would also have required appropriate support from the national level, which, although not discussed in this paper, was itself inappropriately focused on operational control, through mechanisms such as accreditation, rather than on its strategic role in building provincial political support and programme capacity.

## Conclusions

Scaling up debates often highlight considerations of systems capacity, in particular financial and human resource constraints. If these are in place, implementation is often assumed to flow in a more or less linear and hierarchical fashion from cost-effective interventions, elaborated plans and adequate budgets. However, implementation is also a managerial process requiring the ability to design systems, balance core rules with local flexibility, the development of appropriate partnerships and the fostering of political support. Operational research on appropriate programme choices and on how to build managerial capacity, particularly in decentralised health systems, could play an important role in supporting scale up processes.

## Competing interests

The authors declare that they have no competing interests

## Authors' contributions

HS conceived of the analysis, conducted the review and drafted the article. DC designed the models of care studies that formed the basis of the analysis, DVR contributed specialist understanding of the Free State, and LG provided the conceptual framing. All authors read and commented on drafts of the article and approved the final version.

## Supplementary Material

Additional file 1 Operational and strategic aspects of ART programme management in three provinces Click here for file
