# Supplementary material for: Differences in antiretroviral scale up in three South African provinces: the role of implementation management
Source: BMC Health Serv Res. 2010 Jul 2;10(Suppl 1):S4. doi: 10.1186/1472-6963-10-S1-S4 (PMC2895748; doi:10.1186/1472-6963-10-S1-S4)
Supplement: Additional file 1 — Operational and strategic aspects of ART programme management in three provinces [file 1472-6963-10-S1-S4-S1.docx]

## Operational and strategic aspects of ART programme management in three provinces

| **Implementation management tasks** | | **Free State** | **Gauteng** | **Western Cape** |
| --- | --- | --- | --- | --- |
| *Operational management tasks* | | | | |
| Access | Treatment sites per million PSDP* in 2006 | 6.4 | 5.7 | 10 |
|  | Staffing of ART sites (see Figure 2) | High nurse/low doctor : patient ratios | Moderate nurse/moderate doctor : patient ratios | Moderate nurse/high doctor: patient ratios |
|  | Training | Extensive and well organised | | |
| Supply chain management | Drug supplies | Early delays while national tender finalised, availability good subsequently (until 2008/9) | Initiated own procurement while waiting for national tender, availability good | |
| Chronic care systems | Adherence management | Comprehensive approach to treatment preparation and support across ART programme sites | | |
|  | Team work | Multi-disciplinary involvement in treatment preparation and initiation across all sites | | |
| Monitoring and evaluation | Information system | Standardised but complex and poor turnaround times | Weak, non standardised | Standardised, simple and timely production of information |
| *Strategic management tasks* | | | | |
| Provincial Leadership | Political | Not a key feature | HIV Programme driven personally by provincial Premier | ART programme backed by non-ANC party in power |
|  | Managerial/  Administrative | Senior management support but not sustained; Weak/ high turnover of middle management | Middle management structures in districts | Strong leadership from senior management |
| Resource mobilisation | Additional funding (apart from national conditional grant) | None | Provincial allocation from equitable share ** | Successful Global Fund application in 2004 + provincial allocation from equitable share** |
| Programme design | Involvement of clinicians/front-line providers | Formal, but limited to academic hospital | Informal, but extensive through mainly PEPFAR partners | Formal, through a number of academic/NGO partnerships |
|  | Presence of sites prior to 2004 | None | In academic centres | In both academic centres and PHC settings |
|  | Flexibility and adaptation | Standardised provincial model, little flexibility | Some flexibility within national templates | Flexible with standardised provincial information system |
|  | Vertical vs horizontal implementation | Programme based in both primary health care and hospital facilities, but with ring-fenced resources and staffing and limited integration with other services across all sites | | |
| Programme implementation | Coordination within DOH | ART programme became fairly isolated with time | ART programme able to leverage cooperation from other departmental divisions | Senior manager-clinician-NGO partner networks |
|  | Early involvement of district managers | No | Yes | No |
|  | Programme partnerships | Focused on operational research and training. Absence of clinical/service delivery partnerships | Clinical, training and service delivery partnerships, mainly through PEPFAR partners | Clinical, training and service delivery partnerships through a range of partners |
|  | Mechanisms of joint government-civil society governance | Absent to weak | Ad hoc | Joint decision making processes with clinicians and NGO partners |

* PDSP: Public Sector Dependent Population. Source: National Department of Health. 2006. Database of ART facilities in South Africa (dated 20/09/06)

** Provincial equitable share = provincial budget
